# Identification of pyroptosis-related signature for cervical cancer predicting prognosis

**DOI:** 10.18632/aging.203716

**Published:** 2021-11-27

**Authors:** Cankun Zhou, Chaomei Li, Yuhua Zheng, Xiaochun Liu

**Affiliations:** 1 Department of Gynecology, Southern Medical University Affiliated Maternal and Child Health Hospital of Foshan, Foshan 528000, Guangdong, China; 2 Department of Maternity Centre, Southern Medical University Affiliated Maternal and Child Health Hospital of Foshan, Foshan 528000, Guangdong, China

**Keywords:** cervical cancer, pyroptosis, prognosis, tumor microenvironment, immune infiltration

## Abstract

Cervical cancer (CC) is one of the most common malignancies encountered in gynecology practice. However, there is a paucity of information about specific biomarkers that assist in the diagnosis and prognosis of CC. Pyroptosis is a form of programmed cell death whose different elements are related to the occurrence, invasion, and metastasis of tumors. However, the role of pyroptosis phenomena in the progression of CC has not yet been elucidated. This study focuses on the development of a pyroptosis-associated prognostic signature for CC using integrated bioinformatics to delineate the relationships among the signature, tumor microenvironment, and immune response of the patients. In this respect, we identified a prognostic signature that depends on eight pyroptosis-related genes (PRGs) that designate with better prognostic survival in the low-risk group (P<0.05) and where AUC values were greater than 0.7. A multi-factor Cox regression analysis indicated that such a signature could be used as an independent prognostic factor, and both the DCA and the Nomogram suggested that the proposed prognostic signature had good predictive capabilities. Interestingly, this prognostic signature can be applied to multiple tumors and thus, is versatile from a clinical point of view. In addition, there were significant differences in the tumor microenvironment and immune infiltration status between the high- and low-risk groups (P<0. 05). The core gene granzyme B (GZMB) was screened and the CC-associated regulatory axis, GZMB/ miR-378a/TRIM52-AS1, was constructed, which may promote CC progression, and further experimentation is needed to validate these results.

## INTRODUCTION

Cervical cancer (CC) is the fourth most common type of malignancy that affects women across the world [1]. While the incidence and mortality rates of cervical cancer have declined significantly in high-income countries, the overall prognosis for CC patients in low-income countries remains poor [2, 3]. And in the event of recurrence and metastasis, the patient’s prognosis is further reduced, so cervical cancer will remain a heavy burden of disease for a long time to come. Therefore, obtaining a lucid understanding of the molecular mechanisms that underly the development of cervical cancer is crucial, and formulating methods for the early diagnosis and treatment is essential to improving the survival rate of CC patients.

Cell death is mainly divided into two categories, namely programmed death and non-programmed death. Programmed death mainly includes apoptosis, pyroptosis, and necroptosis, and non-programmed death mainly includes cell necrosis. Pyroptosis is a programmed cell death mechanism that is distinct from apoptosis and necrosis. It is mediated by the enzyme called caspase and inflammasomes, and is characterized by a rapid plasma membrane rupture followed by the release of cellular contents and pro-inflammatory substances such as interleukins (IL). This triggers an inflammatory cascade that ultimately destroys the cell [4]. Pyroptosis not only plays an important role in infectious diseases, cardiovascular diseases, and diseases that affect the central nervous system, but is also closely related to the development of tumors, for which it has both favorable and deleterious aspects [5]. On the one hand, when it is considered as an intrinsic immune mechanism, pyroptosis is a process that inhibits the occurrence and development of tumors. On the other hand, when considered as a type of pro-inflammatory cell death, pyroptosis provides a suitable microenvironment for tumor growth. The key elements that are involved in pyroptosis, such as inflammatory vesicles, gasdermin proteins, and pro-inflammatory cytokines, are also associated with the occurrence, invasion, and metastasis of tumors [6, 7]. Meanwhile, earlier studies have found that HeLa cells that overexpress gasdermin–B (GSDMB) exhibit evident pyroptosis characteristics [8]. Immune cells too, promote HeLa pyroptosis by releasing granzyme-B (GZMB) and lysing gasdermin-E (GSDME) [9]. In human papillomavirus (HPV)-positive cells, the expression of IL-1β, an important inflammatory molecule involved in pyroptosis, was completely inhibited. This effectively blocked the containment of tumor cells by pyroptosis. Meanwhile, the expression of SIRT1 in the CC cells affected the stability of the RelB mRNA, which in turn affected the expression of AIM2. When SIRT1 was knocked down, the RelB stability was enhanced significantly and the expression of the inflammation-related AIM2 genes wasup-regulated, triggering AIM2 inflammation vesicle-mediated pyroptosis [10, 11]. However, the characterization and determination of the prognostic value of PRGs for CC has not yet been completed.

In this study, we analyzed the genetic data of cervical cancer in the TCGA (The Cancer Genome Atlas) database and the UCSC Xena database to describe the expression levels and genetic changes of PRGs, and constructed and validated a prognostic model based on CC samples, and screened out core genes and pan-cancer analysis.

## MATERIALS AND METHODS

### Datasets and preprocessing

The TCGA database (https://portal.gdc.cancer.gov/) and the UCSC Xena database (https://xena.ucsc.edu/) were used to obtain CC data. Our research program was shown in Supplementary Figure 1. The RNA sequence data, clinicopathological parameters, and genomic mutation data (including somatic mutations and copy number variants (CNV)) were obtained for 306 CC tissues and 3 paraneoplastic tissue samples. Based on earlier studies [9, 12, 13], 35 PRGs were included, namely AIM2, CASP1, CASP3, CASP4, CASP5, CASP6, CASP8, CASP9, ELANE, GPX4, GSDMA, GSDMB, GSDMC, GSDMD, GSDME, GZMA, GZMB, IL18, IL1B, IL6, NLRC4, NLRP1, NLRP2, NLRP3, NLRP6, NLRP7, NOD1, NOD2, PJVK, PLCG1, PRKACA, PYCARD, SCAF11, TIRAP, and TNF, as detailed in Supplementary Table 1. Comparison of mRNA expression levels of PRGs in paraneoplastic tissue and CC tissues, and visualization of somatic mutations and CNV in CC tissues by the R software ‘maftools’ and the ‘Rcircos’ package. The Kaplan Meier-Plotter online platform (https://kmplot.com/analysis/) was used to further analyze the survival and prognosis of PRGs [14]. All subsequent statistical analyses were performed via R version 4.0.2. The screening criterion for differential expression of PRGs was P<0.05.

### Establishment of prognostic signature

The data for 304 CC patients were obtained from the TCGA database and used as the training dataset to screen for prognosis-related PRGs and exclude samples with no survival time. We constructed a prognostic signature for the PRGs using a univariate Cox proportional regression analysis and the least absolute shrinkage and selection operator (Lasso) regression, which was stratified according to the risk score (Risk score = EXP _PRGs 1_ × Coefficient_1_ + EXP _PRGs 2_ × Coefficient_2_ + ... + EXP _PRGs n_ × Coefficient_n_). Subsequently, the associated risk score was calculated for each patient in the training dataset. Based on the median score, the patient’s data were grouped as low or high risk. The difference in survival between the two groups was determined using the log-rank test and the Kaplan-Meier curves, while the sensitivity and specificity of the prognostic signature were determined from the ROC curves plotted using the ‘survivalROC’ package. To determine the feasibility and reliability of the proposed prognostic signature, the training dataset of CC patients (N = 304) was randomly divided into test datasets A (N = 152) and B (N = 152) using the ‘Caret’ package. Subsequently, the same statistical methods were used for validating both test datasets.

Then, independent prognostic analyses and decision curve analysis (DCA) were performed to combine the clinical features, and a nomogram was constructed to predict the one-, three- and five-year survival rates of CC patients to assess the prognostic value of the signature.

The TCGA pan-cancer transcriptional expression data and clinical data were obtained from the Xena database that includes 33 cancer types, as detailed in Supplementary Table 2. The prognostic model was applied to the remaining 32 tumors to analyze their prognostic outcomes for the different cancers. The risk score was considered to be significantly associated with CC patient survival when p<0.05.

### Tumor mutation burden, tumor microenvironment, and immune cell infiltration analysis

Tumor mutation burden (TMB) is a biomarker that can predict how a patient might respond to immunotherapy [15]. Differences in TMB between the high and low-risk groups were compared based on original data of somatic mutations in CC patients, and the risk scores and TMB were compared using the Spearman correlation analysis. An ESTIMATE algorithm was used to predict the purity of the CC [16], and the proportion of stromal cells (stromal score) and immune cells (immune score) that have infiltrated the tumor tissue as well as the measured differences in the tumor microenvironment (TME) between the two risk groups were assessed.

Immune cell infiltration data from CC were obtained from the Timer 2.0 database (http://timer.cistrome.org/) [17]. Four algorithms were eventually chosen [18–20], including TIMER, CIBERSORT, QUANTISEQ, and MCPCOUNTER, according to the PRG signature, and the correlation between the immune cell infiltration and risk score under different algorithms was analyzed using Spearman correlation and represented as a bubble plot. In addition, the differences in immune function, HLA gene expression, immune checkpoint, and m6A gene expression were compared for the high- and low-risk groups using the Wilcox test. P < 0.05 was considered to be statistically significant.

### Construction of mRNA-miRNA-lncRNA interaction network

To screen for the relevant genes in the prognostic signature, we searched for protein-protein interactions (PPI) via the String (https://string-db.org/) online database. The degrees of connectivity of these PRGs were also counted. Next, the correlations of genes in the PRG signature were evaluated concerning the clinical feature ‘Grade’. The patients were divided into two subgroups, namely, G1-2 and G3-4, and the Wilcox test was used for their analysis. The genes with the highest connectivity and statistically significant correlation with ‘Grade’ in the signature were designated as the core genes.

Then, pan-cancer differential analysis, survival analysis, and TMB correlation analysis were performed on these core genes that were designated in the previous step. We used boxplots to summarize the overall levels of expression of the core gene in the 33 TCGA cancer samples. Based on 18 types chosen from more than five adjacent normal tissues, a linear mixed-effect model was used to compare and analyze the difference in gene expression between the tumor and the paracancerous tissues. The TMB scores were obtained from UCSC Xena database. Correlation analysis between the core gene expression and TMB was performed using Spearman’s method. A forest plot was drawn to determine whether the expression of the core gene correlated with the patient survival obtained by the univariate Cox analysis.

To construct the mRNA-miRNA-LncRNA interaction network for the core genes, the miRNAs targeted by the core genes were predicted using the TargetScan (http://www.targetscan.org/vert_72/) and miRTarBase (http://mirtarbase.cuhk.edu.cn/) databases. The predicted miRNAs were intersected. Subsequently, the differential expression levels and prognostic values of these miRNAs were analyzed. Based on the prognosis and differentially expressed miRNAs, LncBase Predicted v.2 (https://carolina.imis.athena-innovation.gr/diana_tools/web/index.php?r=lncbasev2/index-predicted) and StarBase (http://starbase.sysu.edu.cn/) were used to predict the LncRNAs that the miRNAs might eventually bind. The differential expression levels and prognostic value of these LncRNAs were analyzed in detail, and on this basis, we constructed the mRNA-miRNA-LncRNA interaction networks. The statistically significant difference was P < 0.05.

### Availability of data and materials

The information of this study is obtained by the TCGA, UCSC Xena, Kaplan Meier-Plotter, Timer 2.0, String, TargetScan, miRTarBase, LncBase Predicted v.2, and StarBasedatabase database. We are grateful to them for the source of data used in our study.

## RESULTS

### Genetic variation profile of PRGs in CC

**Figure 1:**
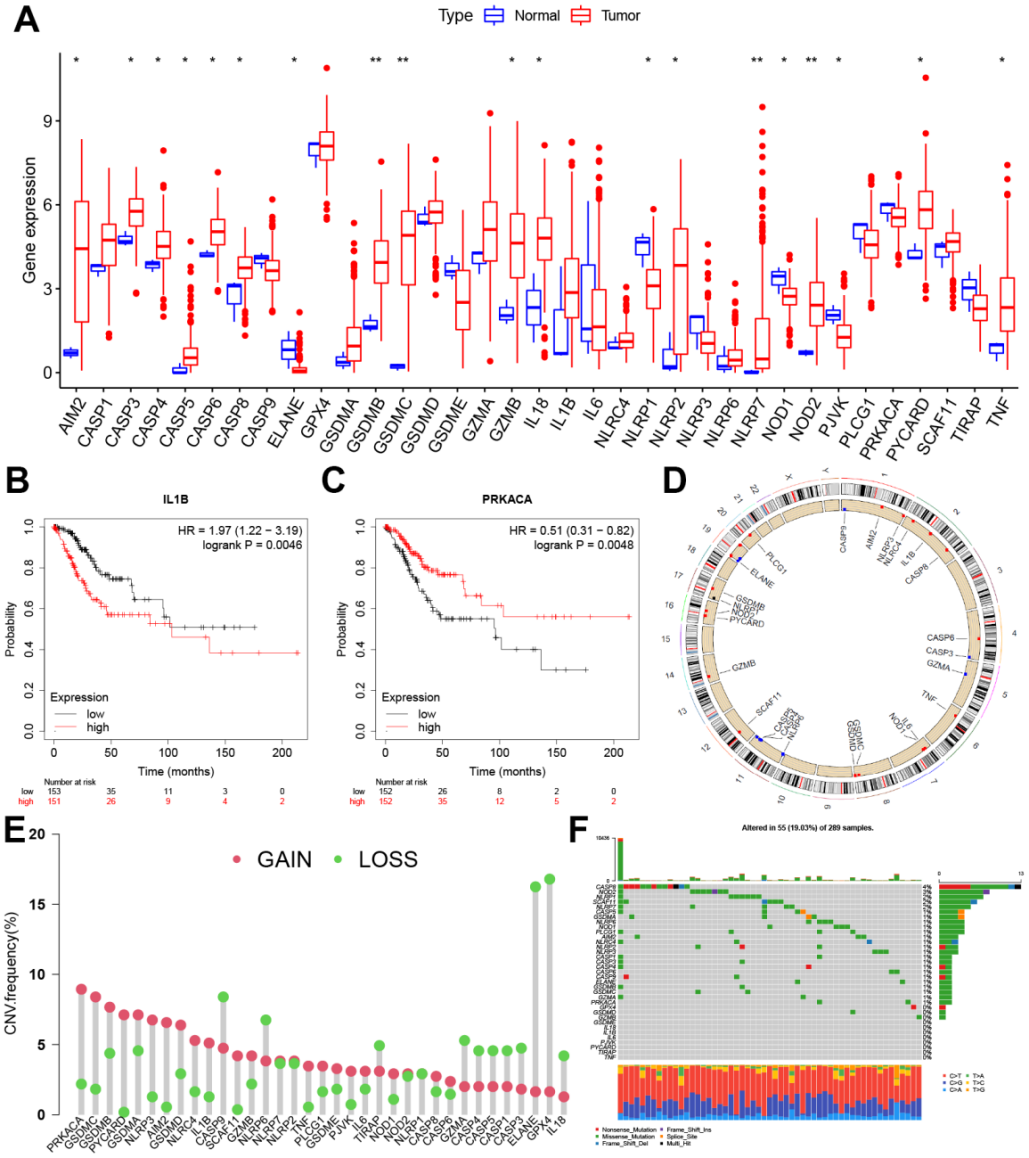
**Expression and genetic variation landscape of PRGs in CC.** (**A**) The boxplot demonstrated the expression of PRGs between normal and CC samples. Patients with low expression of IL 1B (**B**) and high expression of PRKACA (**C**) have more survival benefits. (**D**) Location of CNV alterations in PRGs on 23 chromosomes in CC cohort. (**E**) The CNV variation frequency of PRGs. The red and green dots represent CNV amplification and deletion, respectively. (**F**) Genetic alteration on a query of PRGs. PRGs, Pyroptosis-related genes. CC, Cervical cancer. CNV, Copy number variation. *p < 0.05, **p < 0.01, and ***p < 0.001.

First of all, we found that the AIM2, CASP3, CASP4, CASP5, CASP6, CASP8, GSDMB, GSDMC, GZMB, IL18, NLRP2, NLRP7, NOD2, PYCARD, and TNF were significantly up-regulated in CC tissues, while ELANE, NLRP1, NOD1, and PJVK were significantly down-regulated (Figure 1A). A survival prognosis analysis showed that IL 1B and PRKACA had a significant correlation with the prognosis of CC patients. Those patients with low expression of IL 1B and high expression of PRKACA have a better prognosis for survival (Figure 1B, 1C). Figure 1D shows the locations of the CNV alterations on the PRG chromosomes, and it can be observed that CNV changes were prevalent in these genes. PRKACA had the highest probability of CNV amplification and GPX4 had the highest frequency of CNV deletion (Figure 1E). The somatic mutations of these PRGs in CC were also analyzed and it was concluded that the overall mutation frequency was not high in these cases (≤4%) (Figure 1F).

### Construction and evaluation of the effectiveness of prognostic signature

**Figure 2:**
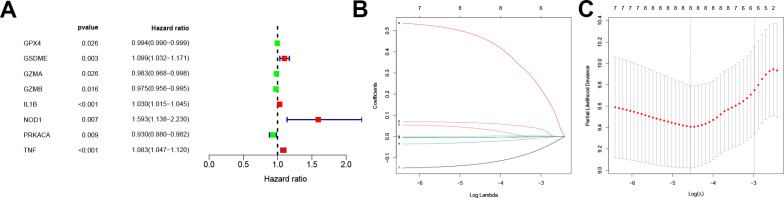
**Screening for prognosis-related PRGs and LASSO regression.** (**A**) Forest plot of the prognosis-related PRGs based on P < 0.05. Red and green indicate high and low risk, respectively. (**B**) LASSO coefficients for PRGs. Each curve represents a PRGs. (**C**) 1,000-fold cross-validation of variable selection in LASSO regressions by 1-SE criteria. PRGs, Pyroptosis-related genes. LASSO, Least absolute shrinkage and selection operator.

A survival analysis of the PRGs in the training dataset was conducted via a univariate Cox proportional regression model and screened for eight PRGs that had prognostic value (Figure 2A). Among these, GZMB and TNF were expressed in high level in CC tissue while NOD1 was significantly low (Supplementary Figure 2B).

**Figure 3:**
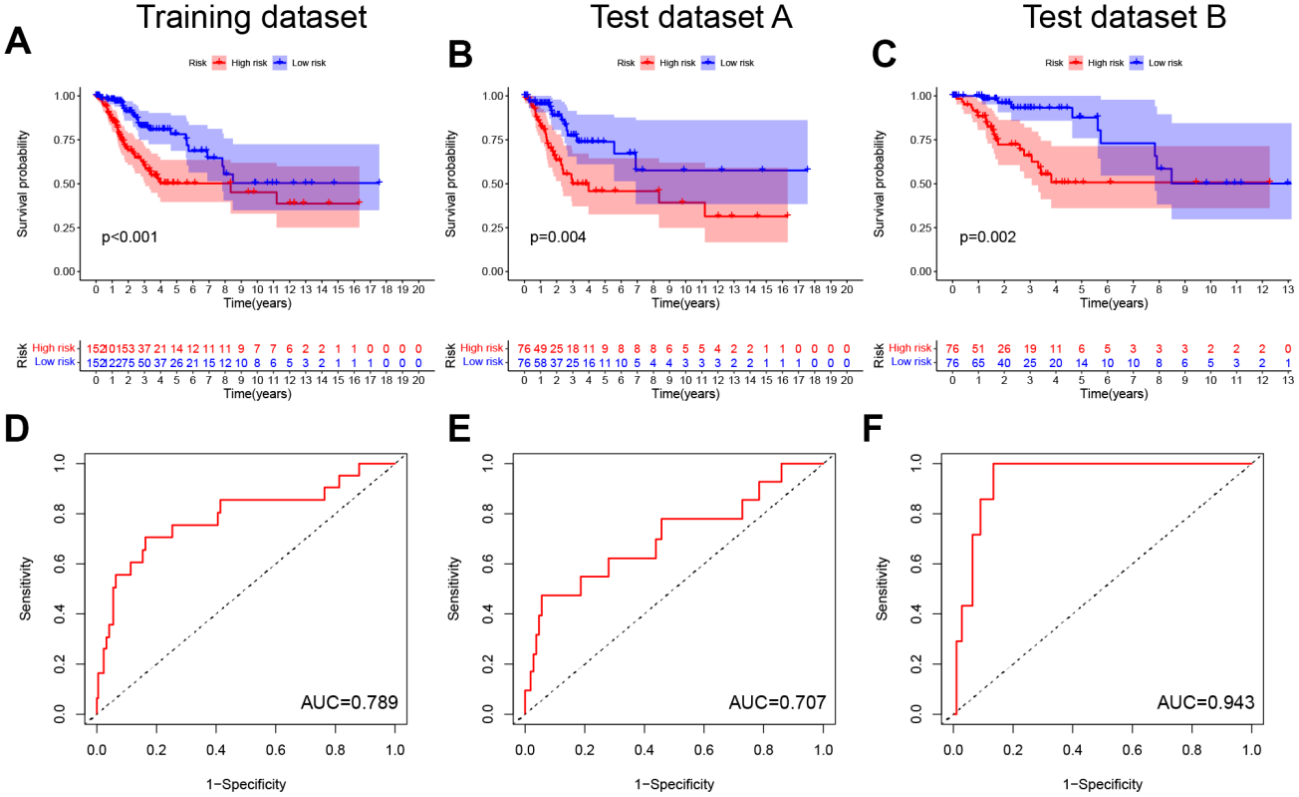
**Construction and validation of the pyroptosis-related signature.** (**A**–**C**) Kaplan-Meier curves showed lower overall survival rates in the high-risk group than in the low-risk group in the training dataset, test dataset A and test dataset B (P < 0.05). (**D**–**F**) The predicted ROC curves for the training dataset, test dataset A and test dataset B were 0.789, 0.707, and 0.943 respectively.

Following this, the pyroptosis-related signature was constructed by further downscaling the prognosis-related PRGs via Lasso regression (Figure 2B, 2C). The risk score was calculated for each patient using the expression, risk score= (-0.0037×EXP_GPX4_) + (0.0377×EXP_GSDME_) + (-0.0043×EXP_GZMA_) + (-0.026×EXP_GZMB_) +(0.0017×EXP_IL1B_) + (0.4741×EXP_NOD1_) + (-0.1298×EXP_PRKACA_) + (0.0642×EXP_TNF_). According to the median, the patients were divided into low-risk and high-risk groups, and the survival prognostic analysis showed a significant survival benefit for patients classified as low-risk (P < 0.001, Figure 3A), with an AUC value of 0.789 (Figure 3D). In test datasets A and B, the difference in survival prognosis (P < 0.05, Figure 3B, 3C) and predictive efficacy (AUC values of 0.707 and 0.943, respectively, Figure 3E, 3F) were validated similarly, thereby confirming the overall accuracy and validity of the prognostic signature. Interestingly, this prognostic model indicated a better prognosis for patients in the low-risk group than in the high-risk group in glioblastoma multiforme (GBM), brain lower grade glioma (LGG), liver hepatocellular carcinoma (LIHC), and uterine corpus endometrial carcinoma (UCEC) (Supplementary Figure 3).

**Figure 4:**
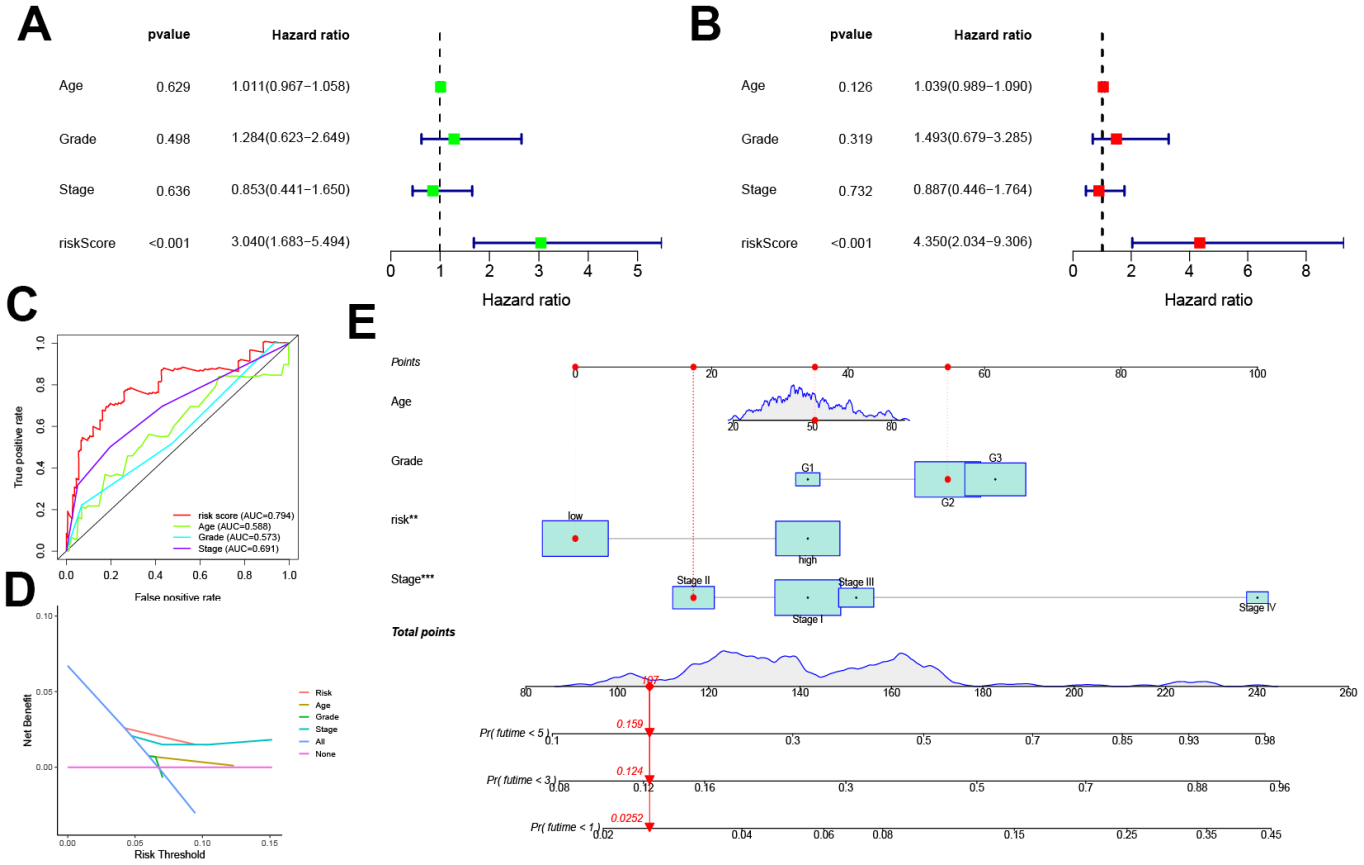
**Prognostic value of the pyroptosis-related signature.** (**A**, **B**) Univariate and multivariate COX analysis for the prognostic signature and clinical features (including Age, Stage, and Grade). (**C**) The AUC values of the prognostic signature and clinical features. (**D**) The DCA of the prognostic signature and clinical features. (**E**) Nomogram for both the signature and clinical features to predict one-, three- and five-year survival rates.

Univariate and multivariate COX analyses in conjunction with the patient age, stage, and grade, showed that this pyroptosis-related signature was an independent prognostic factor for OS in CC patients (p < 0.001, Figure 4A, 4B). In addition, the risk score corresponded to the highest AUC value (AUC = 0.794, Figure 4C) compared to other clinical features. The DCA further demonstrated the clinical usefulness of the signature (Figure 4D). The Nomogram for both the signature and clinical features was stable and accurate, and it can be used to predict the one-year, three-year, and five-year survival rates in CC patients (Figure 4E).

### Correlation of TMB, TME, and immune cell infiltration with prognostic signature

**Figure 5:**
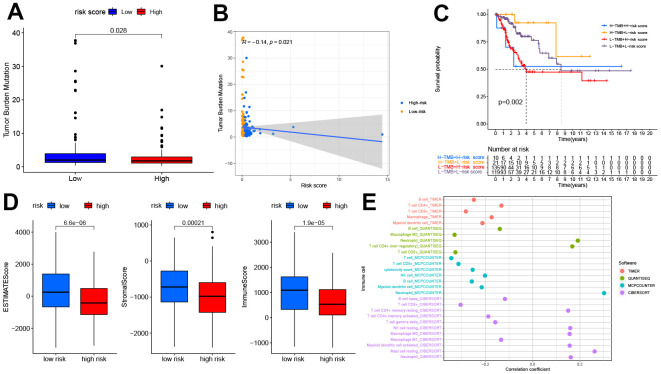
**The relationship between the pyroptosis-related signature and the TMB, TME, and immune cell infiltration.** (**A**) Patients in the high-risk group had lower TMB (P<0.05). (**B**) The correlation between risk score and TMB (R=-0.14, P<0.05). (**C**) Kaplan-Meier curves showed lower overall survival rates in the low-TMB combined with the high-risk group than in the other three groups(P<0.05). (**D**) The relationship between the risk score and TME, patients in the high-risk group had the lower stromal score, immune score, and estimate score(P<0.05). (**E**) The relationship between the risk score and immune cell infiltration is based on TIMER, QUANTISEQ, MCPCOUNTER, and CIBERSORT algorithm. Bubble plot for immune responses significantly associated with a risk score. TMB, tumor mutation burden. TME, tumor microenvironment.

Previous studies have indicated the crucial role of TME in the occurrence and development of tumors [21]. As TMB predicts patient response to immunotherapy, our study found that patients in the high-risk group typically had low TMB scores, and the risk score was negatively correlated with the TMB score to a significant extent. The results of the survival analysis showed that patients that scored high on risk and low on TMB possessed a significant survival advantage (Figure 5A–5C). Compared to the high-risk group, the ESTIMATEScore, ImmuneScore, and StromalScore were all higher in the low-risk group (Figure 5D).

**Figure 6:**
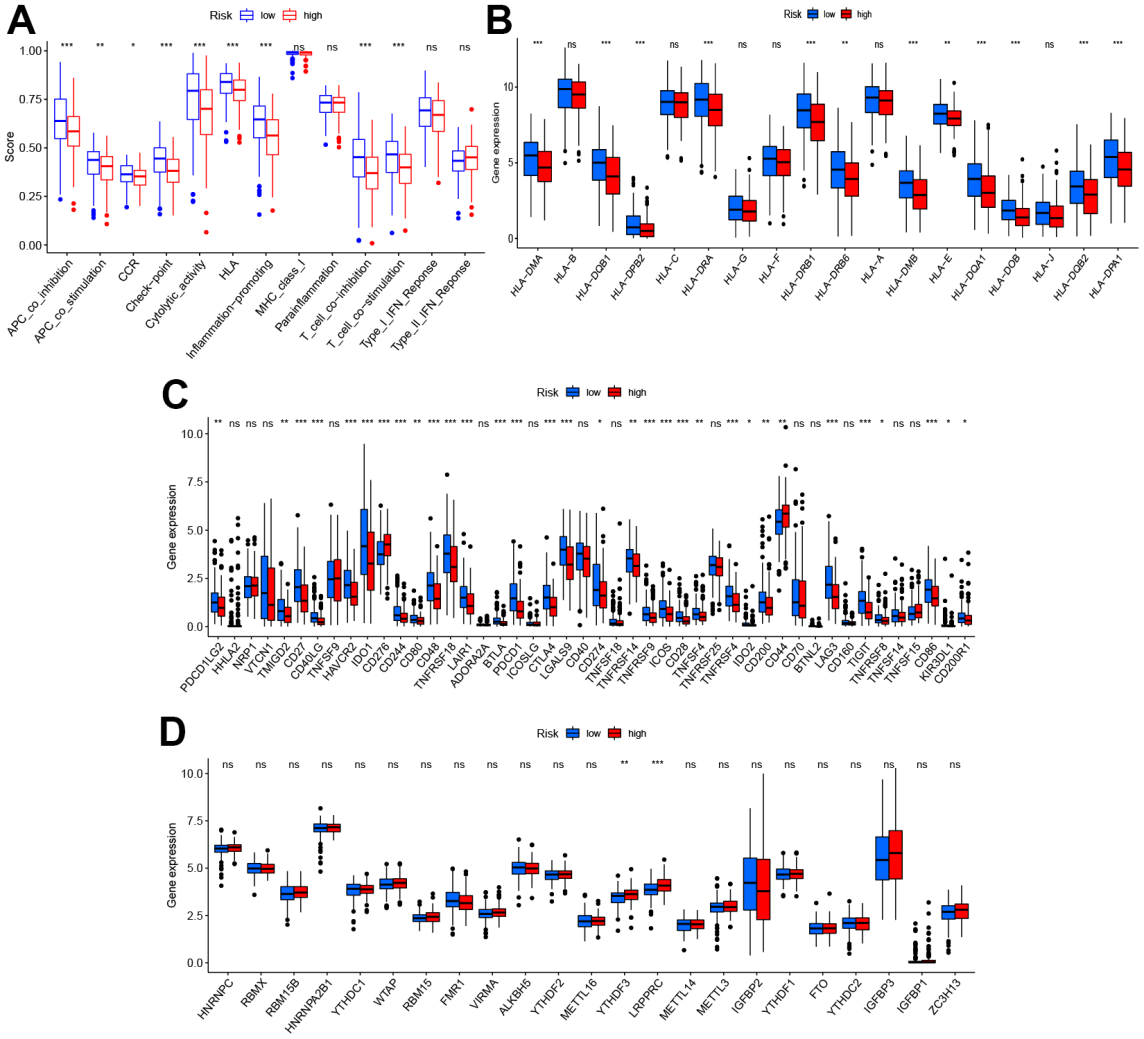
**Diversity of immune microenvironment characteristics in high- and low-risk groups.** (**A**) The activity differences of each immune function between high and low-risk groups. (**B**) The differences of each HLA-related gene between both risk groups. (**C**) The expression status of immune checkpoints between both risk groups. (**D**) The expression status of m6A-related genes between both risk groups. *p < 0.05, **p < 0.01, and ***p < 0.001.

The relation between the immune response and risk score based on the TIMER, QUANTISEQ, MCPCOUNTER, and CIBERSORT algorithms is shown in Figure 5E. The risk score was negatively correlated to the infiltration levels of B cells, T cell CD8+, and macrophages, and positively correlated to the infiltration level of neutrophils. The analysis of immune function differences showed that mechanisms such as the antigen-presenting cell (APC) co-inhibition/ APC_co_stimulation, CC chemokine receptor (CCR), check-point, cytolytic_activity, human leukocyte antigen (HLA), Inflammation promotion, and T cell co-inhibition/ stimulation were significantly more active in the low-risk group (Figure 6A).

Considering this analysis of the immune function, the levels of HLA-related gene expression l were further studied as shown in Figure 6B. The levels of expression of HLA-DMA, HLA-DQB1, HLA HLA-DMA, HLA-DQB1, HLA-DRA, etc. were significantly higher in the low-risk group. As checkpoint inhibitors are crucial to immunotherapy, the differences in immune checkpoint expression between the two groups were analyzed in detail. Significant differences were demonstrated in the expression of PDCD1LG2, TMIGD2, CD27, CD40LG, etc. for the two groups (Figure 6C). Recent research suggests that m6A can regulate the immune response and also modify the immune microenvironment of the tumor [22]. On comparing the m6A-related expression of mRNA between the high- and low-risk groups, it was seen that YTHDF3 and LRPPRC ad significantly high levels of expression in the high-risk group (Figure 6D).

### Construction of mRNA-miRNA-lncRNA interoperability network

The interrelationships between the 35 PRGs considered in this study were retrieved from the STRING database and a PPI network having a composite score of > 4.0 was constructed. A complex, close-knit network of interrelationships was found among these genes was determined (Supplementary Figure 4A). Subsequently, the degree of connectivity between these genes (Supplementary Figure 4B). We further examined the relationship between levels of mRNA expression in the pyroptosis-related signature and CC grade staging, and found that the expressions of GPX4, GZMA, and GZMB were significantly higher in the G3-4 group compared to the G1-2 group (P < 0.05, Supplementary Figure 4C–4F). Moreover, the degree of connectivity of GPX4, GZMA, and GZMB in the PPI network above was 4, 4, and 10, respectively, suggesting that GZMB may indeed be involved in tumor growth and progression in CC patients.

**Figure 7:**
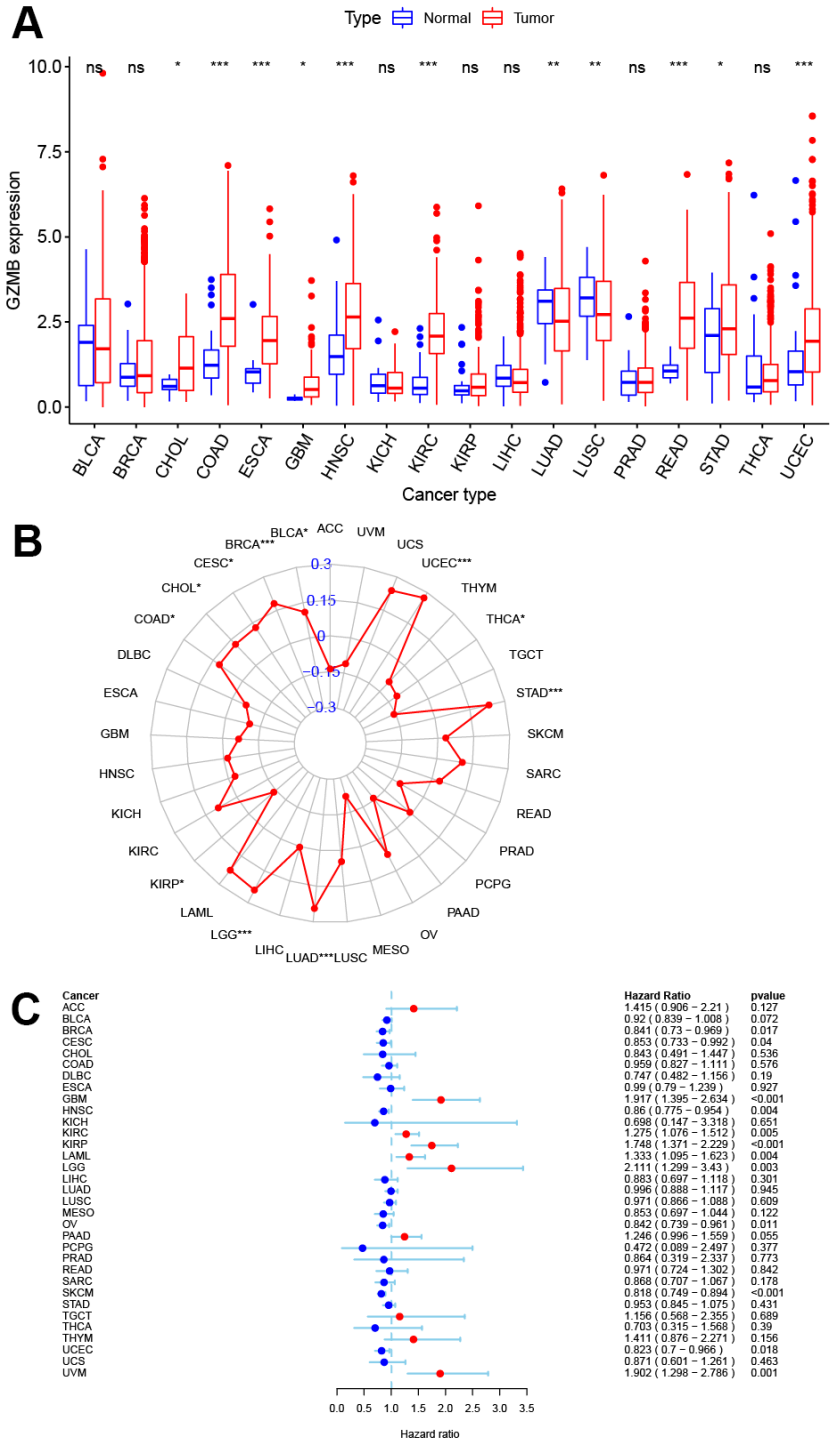
**Pan-cancer analysis of core gene GZMB expression and survival prognosis.** (**A**) Boxplot of GZMB differential expression between cancer and adjacent normal tissues. (**B**) Radar graph indicating the correlation between the GZMB expression and TMB in pan-cancer by Spearman’s method. (**C**) Expression of GZMB correlates with overall survival in patients with different cancer types using univariate Cox proportional hazard regression models. *p < 0.05, **p < 0.01, and ***p < 0.001.

Pan-cancer expression analysis was performed for GZMB, which showed its significantly high expression in cholangiocarcinoma (CHOL), colon adenocarcinoma (COAD), esophageal carcinoma (ESCA), glioblastoma multiforme (GBM), head and neck squamous cell carcinoma (HNSC), kidney renal clear cell carcinoma (KIRC), rectum adenocarcinoma (READ), stomach adenocarcinoma (STAD), and UCEC tissues, while significantly low expression was observed in lung adenocarcinoma (LUAD) and lung squamous cell carcinoma (LUSC) tissues (Figure 7A). TMB is a biomarker that indicates response to immunotherapy, and so, the relation between the expression of GZMB and TMB was investigated. The levels of expression of GZMB were significantly and positively correlated with TMB in bladder urothelial carcinoma (BLCA), breast invasive carcinoma (BRCA), cervical squamous cell carcinoma, and endocervical adenocarcinoma (CESC orCC), CHOL, COAD, LGG, LUAD, STAD, and UCEC, Meanwhile, the correlation was significantly negative with kidney renal papillary cell carcinoma (KIRP) and thyroid carcinoma (THCA) (Figure 7B). Interestingly, the cases of CHOL, COAD, STAD, and UCEC in the high-GZMB-expression group also had high TMB values, and these are expected to be more sensitive to immunotherapy. In contrast, LUAD in the low expression group had low TMB values and so, may be resistant to cell scorch-related immunotherapy. Prognostic correlation analysis showed that the GZMB was negatively correlated with survival in BLCA, BRCA, CESC, ovarian serous cystadenocarcinoma (OV), skin cutaneous melanoma (SKCM), UCEC, and uveal melanoma (UVM). Meanwhile, it was positively correlated with GBM, KIRC, KIRP, acute myeloid leukemia (LAML), and LGG (P<0.05, Figure 7C).

**Figure 8:**
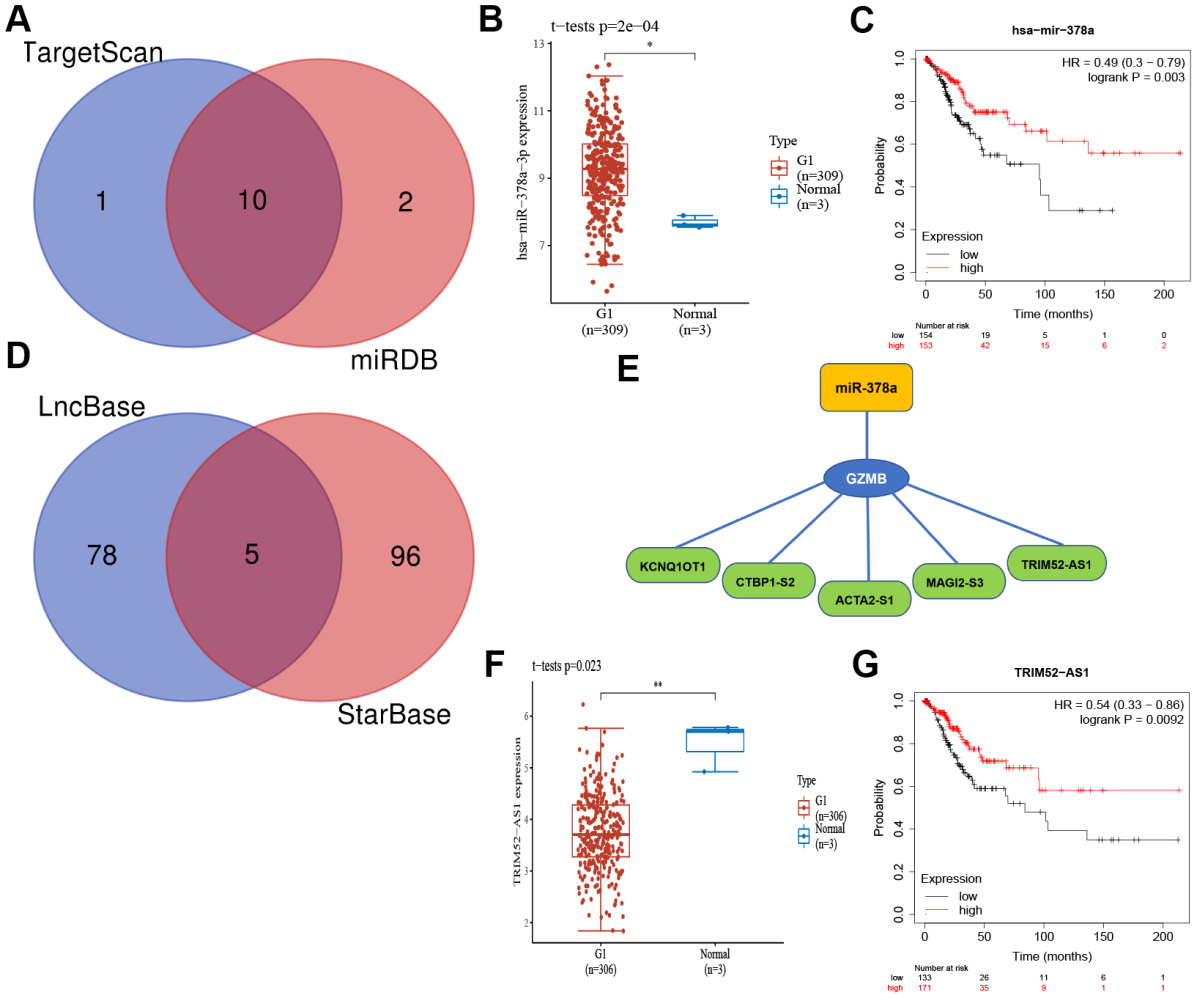
**The core gene GZMB associated mRNA-miRNA-lncRNA interaction network construction.** (**A**) Venn diagram of miRNAs of TarBase and mirTarBase. The expression (**B**) and prognostic value (**C**) of miR-378a (Synonyms: has-miR-378a-3p) in CC patients. (**D**) Venn diagram of LncRNAs of LncBase and StarBase. (**E**) Regulatory network mRNA-miRNA-LncRNA. The expression (**F**) and prognostic value (**G**) of TRIM52-AS1 in CC patients. CC, Cervical cancer.

To elucidate the potential molecular regulation mechanism of GZMB in CC, we further constructed an mRNA-miRNA-LncRNA interaction network. First, based on the comprehensive prediction using the TarBase and mirTarBase databases, we screened out miRNAs that can target and regulate the GZMB core gene, and subsequently, ten miRNAs were obtained by considering the intersection (Figure 8A). Further analysis showed that miR-378a was significantly upregulated in CC (Figure 8B) and patients who show a high expression have a better prognosis for survival (Figure 8C). The LncRNA targets upstream of miR-378a were obtained from the LncBase and StarBase databases, and a total of five LncRNAs (Figure 8D) were obtained by intersection to finally construct the mRNA-miRNA-LncRNA interaction network (Figure 8E). Subsequently, these five LncRNAs were subjected to differential expression analysis and survival prognosis analysis, which showed that TRIM52-AS1 was significantly downregulated in CC (Figure 8F), and its high level of expression indicated a considerable survival advantage (Figure 8G). Thus, it may be concluded that the GZMB/miR-378a/TRIM52-AS1 regulatory axis plays an important regulatory role in the development of CC.

## DISCUSSION

Pyroptosis is a type of programmed cell death that plays a dual role in the occurrence and development of tumors. It can regulate cell morphology, proliferation, infiltration, migration, and chemotherapy resistance through a variety of cell signaling pathways, thereby influencing tumor progression, and may be associated with patient prognosis [5]. However, the regulatory mechanisms and networks that link pyroptosis and CC are not fully understood. Our study aims to elucidate this aspect and paths that can be used for developing interventions.

In terms of gene expression, we first found that genes AIM2, CASP3, CASP4, CASP5, CASP6, CASP8, GSDMB, GSDMC, GZMB, IL18, NLRP2, NLRP7, NOD2, PYCARD, and TNF were chiefly up-regulated in CC tissues, while the genes ELANE, NLRP1, NOD1, and PJVK were down-regulated. Among these, those CC patients who had low IL 1B expression and high PRKACA expression had poorer survival rates. This is consistent with earlier findings that indicate that these PRGs are closely associated with tumor development. In cervical, gastric, and colorectal cancers, high IL-1B expression has been associated with shorter patient survival [23, 24]. In addition, it has been noted that IL-1β expression was elevated in prostate cancers that show a high tendency to metastasize [25]. During theirstudy of head and neck tumors, Dong et al. found a considerable increase in lymph node metastasis with increased IL-1β expression [26]; this indicated that high IL1B expression is detrimental to the survival of tumor patients. Many studies have shown that PRKACA is crucial to the development and progression of a variety of cancers [27], and also that it can be used as a biomarker for hepatocellular carcinoma [28]. Therefore, these genes have the potential to be new molecular targets for CC.

In ovarian and gastric cancers, researchers have identified PRGs and also constructed and validated several prognostic models for them [13, 29]. Hence, a pyroptosis-related signature was formulated, which consisted of eight PRGs with prognostic value (GPX4, GSDME, GZMA, GZMB, IL1B, NOD1, PRKACA, TNF); the univariate Cox proportional risk regression and Lasso regression were used, where patients in the low-risk group had better prognosis of survival. The signature could effectively predict the prognosis for CC patients (AUC > 0.7), and several independent prognostic analyses have shown that this signature can be considered as an independent prognostic factor. The associated nomogram has shown to be stable and accurate, and therefore can be used satisfactorily to predict survival at one, three, and five years in CC patients, presenting additional options for prognosis prediction in CC.

Another important finding indicates that this prognostic model has a significant relation with the TME, which confirms that pyroptosis plays a notable role in the TME. A mounting body of evidence shows that cell death by pyroptosis is particularly important for the formation of tumors and TME, and it has a more marked effect on the immune microenvironment of the tumor [30]. Studies have confirmed that approximately 95% of cervical cancer cases may be caused by persistent HPV infections. Consequently, the human immune system plays an important role in the body’s infection response process, thus laying the foundation for the immunotherapy of cancer [31].

This was confirmed in our study, which determined significant differences in TME and immune function between the low- and high-risk groups. It was found that patients with high TME scores had a better prognosis. This finding is consistent with previous studies that concluded that patients with high TME scores exhibited a stronger anti-tumor immune response, stood to benefit more from immunotherapy, and survive longer [32, 33]. In addition, the correlation analysis showed that the B cell and T cell CD8+ correlated negatively with the risk scores, while neutrophils correlated positively with them. Recent studies have shown that the gene GSDME is a tumor suppressor that can enhance the phagocytosis of tumor-associated macrophages and promote the infiltration and activation of NK cells and CD8+ T lymphocytes, thereby inhibiting tumor growth [9]. Xi et al. found that GSDMD was positively correlated with the expression levels of CD8A, GZMB, and IFNG in nonsmall cell lung cancer (NSCLC) tissues, and the expression was up-regulated in activated CD8+ T cells. This enhanced the lethality of CD8+ T cells towards the NSCLC cells [34]. Consistent with this result, Wang et al. found that when a bio-orthogonal shear system was applied to gasdermin protein activated pyroptosis, it reshaped the tumor immune microenvironment of the tumor and activated a strong T cell-mediated anti-tumor immune response that exerted a powerful anti-tumor effect [35]. It was also observed that tumor-associated neutrophils are involved in regulating tumor development, while IL-1β and IL-18 route neutrophils to tumor sites [36]. Meanwhile, GSDMD induces neutrophil death [37]. Neutrophils may be involved in the local and systemic triggering of immune escape in cervical cancer cells. An increase in the total neutrophil count in progressive cervical cancer reduces the anti-tumor activity of T cells and suppresses thereby suppressing the immune action [38–40]. At the same time, our study confirmed that the expression of multiple immune checkpoints and m6A-related mRNAs showed considerable difference between the low- and high-risk groups. Later studies have found that the abnormal expression of the immune checkpoints affects the immune microenvironment of the tumor and helps tumor cells evade the body’s immune response [41, 42]. These studies unequivocally indicate that the host immune response and microenvironment are closely related to the progression of CC.

In our study, GZMB, which is a component of the prognostic model, was significantly upregulated in CC tissues. That is, GZMB expression was significantly up-regulated in patients with advanced (grade 3 and grade 4) CC, while it was negatively associated with survival. GZMB is a serine protease and a pro-inflammatory molecule that promotes the progression of inflammatory diseases and cancers [43]. It is expressed in uroepithelial carcinoma, pancreatic cancer, and melanoma cells, and is known to promote cancer cell invasion [44–46]. Our study also found that GZMB was negatively associated with survival in both BLCA and SKCM. For a long time, GZMB has been established as being influential in protein hydrolysis-mediated apoptosis, while its role in pyroptosis has not been given due attention. GZMB was found to be able to cleave GSDME and kill lymphocytes to activate the process of pyroptosis, thus changing apoptosis to pyroptosis [9].

The GZMB/miR-378a/TRIM52-AS1 regulatory axis that has a strong correlation to the development of CC was extracted from the mRNA-miRNA-LncRNA interaction network. MicroRNA-378a (miR-378a, previously known as miR-378) is an important small molecule that belongs to the non-coding RNA family. The aberrant up-regulation of miR-378a in HPV16/18 positive cervical cancer tissues has been reported earlier. The expression of miR-378a may be elevated by the action of oncoprotein E6/E7 [47]. ElevatedmiR-378a expression has also been reported in cervical cancer tissues and cell lines, and may act through the ST7L/Wnt/β-catenin signaling pathway [48]. At present, there is a paucity of investigations regarding the regulation of TRIM52-AS1 in tumors. For example, the down-regulation of TRIM52-AS1 inhibited the proliferation and migration of renal cell carcinoma (RCC) cells and promoted apoptosis in them. Studies on hepatocellular carcinoma (HCC) have shown that the TRIM52-AS1 knockdown inhibits the proliferation and metastasis of HCC cells. TRIM52-AS1 behaves as a competitive endogenous RNA (ceRNA) and promotes the progression of HCC by sponging the miRNAs and up-regulating the expression of the mRNAs that are regulated by miRNAs [49, 50]. Therefore, it can be concluded that TRIM52-AS is down-regulated in CC tissues; this attenuates the sponging effect on miR-378a, and increases the binding of miR-378a to GZMB, which in turn inhibits and down-regulates the expression of GZMB. This agrees with our findings that the overall survival was significantly lower in the group that showed low-TRIM52-AS1 expression than in the group with high-TRIM52-AS1 expression (Figure 8G) in CC patients. Moreover, the lower the expression of GZMB as a protective factor (Figure 7C), the worse the prognosis was; thus, TRIM52-AS1 may be concluded to promote the progression of cervical cancer through miR-378a/GZMB, which needs to be studied further for validation.

In conclusion, we formulated an effective prognostic signature and constructed a GZMB/miR-378a/TRIM52-AS1 regulatory axis based on several PRGs. This is expected to be relevant in the theoretical study of molecular mechanisms and for assessing the prognosis of cervical cancer patients. However, our study has some limitations. Tumor heterogeneity has not been considered in this study although it cannot be overlooked and must be validated by more extensive *in-vivo* and *in-vitro* studies.

## Supplementary Material

Supplementary Figures

Supplementary Tables
