# Supplementary material for: Identification of pyroptosis-related signature for cervical cancer predicting prognosis
Source: Aging (Albany NY). 2021 Nov 27;13(22):24795–814. doi: 10.18632/aging.203716 (PMC8660613; doi:10.18632/aging.203716)
Supplement: Supplementary Figures [file aging-13-203716-s001.pdf]

SUPPLEMENTARY FIGURES

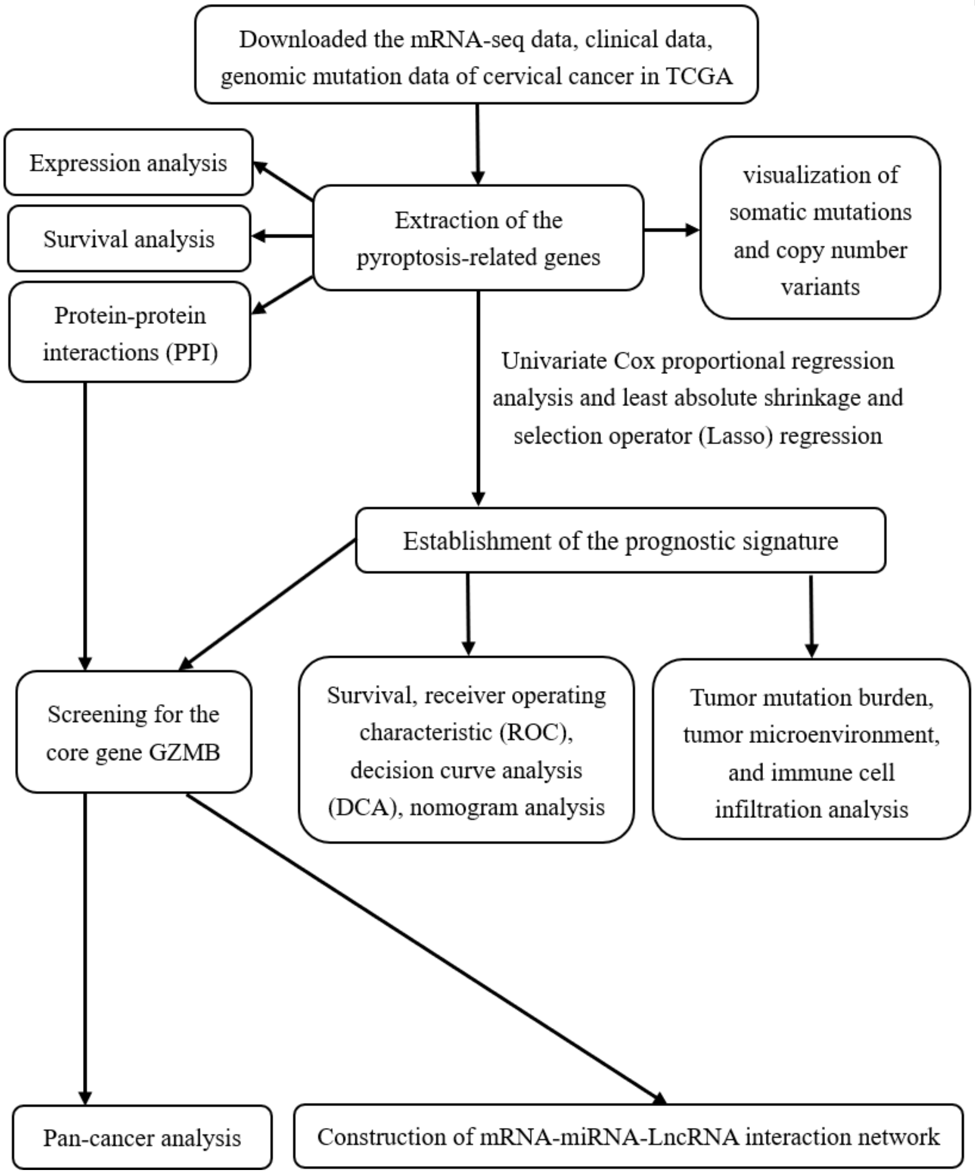

Supplementary Figure 1. Study flow diagram.

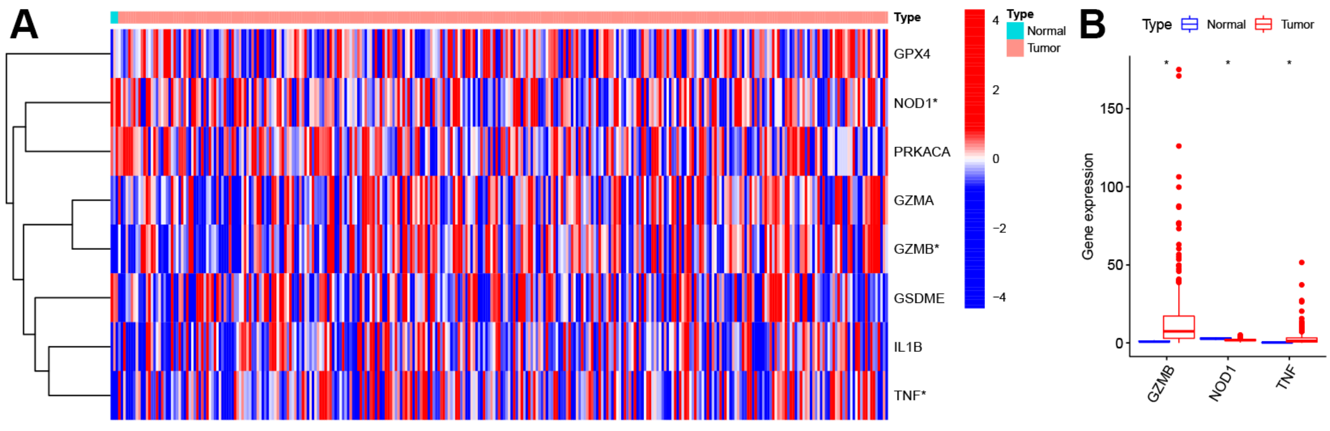

Supplementary Figure 2. Prognostic pyroptosis-related genes. (**A**) Heatmap. (**B**) Boxplot. Compared to normal cervical tissues, GZMB and TNF are highly expressed in cervical cancer tissues, while NOD1 is lowly expressed. *p < 0.05, **p < 0.01, and ***p < 0.001.

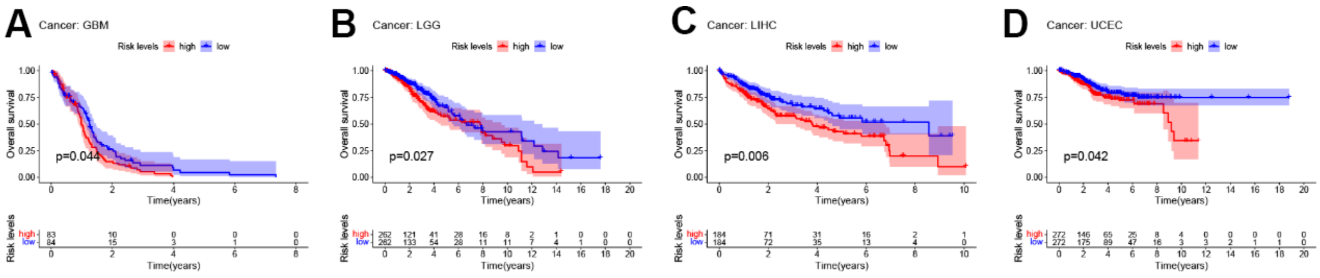

Supplementary Figure 3. The pyroptosis-related signature in other tumors. Kaplan-Meier curves showed lower overall survival rates in the high-risk group than in the low-risk group in GBM (**A**), LCG (**B**), LIHC (**C**), and UCEC (**D**) (P < 0.05). GBM, Glioblastoma multiforme. LGG, Brain Lower Grade Glioma. LIHC, Liver hepatocellular carcinoma. UCEC, Uterine Corpus Endometrial Carcinoma.

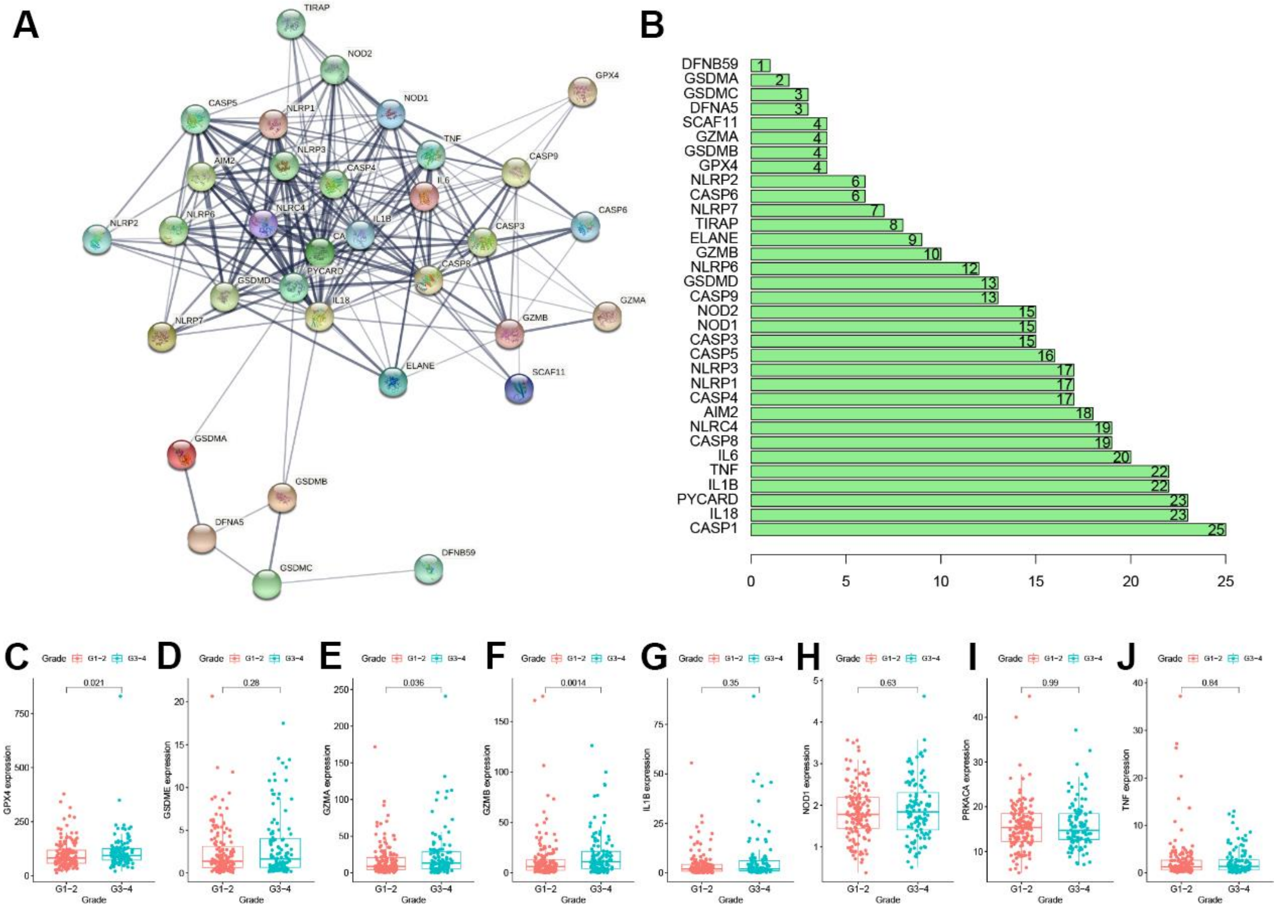

Supplementary Figure 4. Identify the core gene of the pyroptosis-related signature. (**A**) PPI network of the PRGs. (**B**) The degree of binding of PRGs. (**C**–**J**) Correlation of genes of the pyroptosis-related signature with the clinical feature Grade. The expression of GPX4, GZMA, and GZMB was significantly higher in the G3-4 group. PRGs, Pyroptosis-related genes.
