# Supplementary material for: Identification of pyroptosis-related signature for cervical cancer predicting prognosis
Source: Aging (Albany NY). 2021 Nov 27;13(22):24795–814. doi: 10.18632/aging.203716 (PMC8660613; doi:10.18632/aging.203716)
Supplement: Supplementary Tables [file aging-13-203716-s002.pdf]

SUPPLEMENTARY TABLES

Supplementary Table 1. Detailed list of pyroptosis genes.

Gene	Full-names
AIM2	Absent in melanoma 2
CASP1	cysteine-aspartic acid protease-1
CASP3	cysteine-aspartic acid protease-3
CASP4	cysteine-aspartic acid protease-4
CASP5	cysteine-aspartic acid protease-5
CASP6	cysteine-aspartic acid protease-6
CASP8	cysteine-aspartic acid protease-8
CASP9	cysteine-aspartic acid protease-9
ELANE	elastase, neutrophil expressed
GPX4	glutathione peroxidase 4
GSDMA	gasdermin A
GSDMB	gasdermin B
GSDMC	gasdermin C
GSDMD	gasdermin D
GSDME/DFNA5	gasdermin E
GZMA	granzyme A
GZMB	granzyme B
IL18	interleukin 18
IL1B	interleukin 1 beta
IL6	interleukin 6
NLRC4	NLR family CARD domain containing 4
NLRP1	NLR family pyrin domain containing 1
NLRP2	NLR family pyrin domain containing 2
NLRP3	NLR family pyrin domain containing 3
NLRP6	NLR family pyrin domain containing 6
NLRP7	NLR family pyrin domain containing 7
NOD1	nucleotide binding oligomerization domain containing 1
NOD2	nucleotide binding oligomerization domain containing 2
PJVK	pejvakin/deafness, autosomal recessive 59
PLCG1	phospholipase C gamma 1
PRKACA	protein kinase cAMP-activated catalytic subunit alpha
PYCARD	PYD and CARD domain containing
SCAF11/SFRS2IP	SR-related CTD associated factor 11
TIRAP	TIR domain containing adaptor protein
TNF	tumor necrosis factor

Supplementary Table 2. Detailed list of pan-cancer names.

Cancer type	Full-names
ACC	Adrenocortical carcinoma
BLCA	Bladder Urothelial Carcinoma
BRCA	Breast invasive carcinoma
CESC	Cervical squamous cell carcinoma and endocervical adenocarcinoma
CHOL	Cholangiocarcinoma
COAD	Colon adenocarcinoma
COADREAD	Colon adenocarcinoma/Rectum adenocarcinoma Esophageal carcinoma
DLBC	Lymphoid Neoplasm Diffuse Large B-cell Lymphoma
ESCA	Esophageal carcinoma
FPPP	FFPE Pilot Phase II
GBM	Glioblastoma multiforme
GBMLGG	Glioma
HNSC	Head and Neck squamous cell carcinoma
KICH	Kidney Chromophobe
KIPAN	Pan-kidney cohort (KICH+KIRC+KIRP)
KIRC	Kidney renal clear cell carcinoma
KIRP	Kidney renal papillary cell carcinoma
LAML	Acute Myeloid Leukemia
LGG	Brain Lower Grade Glioma
LIHC	Liver hepatocellular carcinoma
LUAD	Lung adenocarcinoma
LUSC	Lung squamous cell carcinoma
MESO	Mesothelioma
OV	Ovarian serous cystadenocarcinoma
PAAD	Pancreatic adenocarcinoma
PCPG	Pheochromocytoma and Paraganglioma
PRAD	Prostate adenocarcinoma
READ	Rectum adenocarcinoma
SARC	Sarcoma
SKCM	Skin Cutaneous Melanoma
STAD	Stomach adenocarcinoma
STES	Stomach and Esophageal carcinoma
TGCT	Testicular Germ Cell Tumors
THCA	Thyroid carcinoma
THYM	Thymoma
UCEC	Uterine Corpus Endometrial Carcinoma
UCS	Uterine Carcinosarcoma
UVM	Uveal Melanoma
